# Microscopic Extrathyroidal Extension Results in Increased Rate of Tumor Recurrence and Is an Independent Predictor of Patient’s Outcome in Middle Eastern Papillary Thyroid Carcinoma

**DOI:** 10.3389/fonc.2021.724432

**Published:** 2021-12-01

**Authors:** Sandeep Kumar Parvathareddy, Abdul K. Siraj, Zeeshan Qadri, Felisa DeVera, Khawar Siddiqui, Saif S. Al-Sobhi, Fouad Al-Dayel, Khawla S. Al-Kuraya

**Affiliations:** ^1^ Human Cancer Genomic Research, Research Center, King Faisal Specialist Hospital and Research Center, Riyadh, Saudi Arabia; ^2^ Department of Pediatric Hematology-Oncology, King Faisal Specialist Hospital and Research Center, Riyadh, Saudi Arabia; ^3^ Department of Surgery, King Faisal Specialist Hospital and Research Center, Riyadh, Saudi Arabia; ^4^ Department of Pathology, King Faisal Specialist Hospital and Research Centre, Riyadh, Saudi Arabia

**Keywords:** microscopic extrathyroidal extension, papillary thyroid carcinoma, recurrence, AJCC staging, recurrence-free survival

## Abstract

**Background:**

Papillary Thyroid Cancer (PTC) is the most common endocrine malignancy, with recurrence rate as high as 30%. A great deal of controversy surrounds the significance of microscopic extrathyroidal extension (m-ETE) as a prognostic factor. The most recent edition (8^th^) of American Joint Committee on Cancer (AJCC) staging system has removed m-ETE from the definition of pT3, which suggests that m-ETE may lack prognostic impact in PTC patients. Moreover, data about m-ETE prevalence and clinical impact on Middle Eastern PTC remains unknown. We therefore investigate the prevalence of m-ETE and its clinico-pathological correlation and prognostic impact in Middle Eastern PTC. We also compared the AJCC 7^th^ and 8^th^ staging systems and their prognostic performance.

**Methods:**

PTCs from 1430 consecutive adult (> 18 years) patients from single tertiary care hospital were included in this study. A retrospective analysis of PTC patients’ survival and recurrence were compared between AJCC 8^th^ and AJCC 7^th^ staging systems using Proportion of Variation Explained (PVE) and Harrell’s C-index.

**Results:**

Median follow up of the study cohort was 9.3 years. 31.2% (446/1430) of patients had m-ETE. In the overall cohort, m-ETE was associated with multiple adverse features such as older age (p < 0.0001), male sex (p = 0.0245), tall cell variant (p < 0.0001), bilateral tumors (p < 0.0001), multifocality (p < 0.0001), lymphovascular invasion (p < 0.0001), lymph node metastasis (p < 0.0001), distant metastasis (p = 0.0166), tumor recurrence (p < 0.0001), radioactive iodine refractoriness (p < 0.0001), *BRAF* mutation (p < 0.0001) and reduced recurrence-free survival (RFS; HR = 1.75; 95% CI = 1.30 – 2.35; p < 0.0001) irrespective of tumor size. Of the 611 patients with T3 disease based on AJCC 7^th^ edition, 359 (58.8%) were down-staged in AJCC 8^th^ edition classification. Overall, the prognostic performance of AJCC 8^th^ edition was inferior to AJCC 7^th^ on the basis of lower PVE (3.04% *vs*. 3.73%) and lower C-index (0.40 *vs*. 0.48).

**Conclusions:**

In Middle Eastern PTC, m-ETE is significantly associated with compromised survival and acts as an independent predictor of RFS. Given these findings, m-ETE should be included in the thyroid cancer treatment guidelines.

## Introduction

The incidence of thyroid cancer has rapidly increased over the past two decades (1, 2). Papillary thyroid cancer (PTC) is the most common thyroid malignancy and generally carries favorable prognosis (3–5), whereby low risk PTC patients have excellent outcome with conservative treatment, such as adequate surgery and TSH suppressive therapy (6, 7). However, a subset of PTC patients present with aggressive disease and experience recurrence, leading to poor prognosis (8–10). Therefore, identifying tumors with potentially aggressive behavior and increased likelihood of recurrence is crucial for therapeutic decision-making and appropriate patient management. Interestingly, PTC is one of the most common cancers in Saudi Arabia and is the second commonest cancer affecting Saudi females (11). Middle Eastern PTCs show a relatively higher rate of recurrence than in Western countries (12–15). Therefore, identifying patients at risk of recurrence is a critical step in the management of high risk PTCs, so that appropriate treatment can be initiated.

A number of prognostic factors, including age, sex, histology, tumor size, vascular invasion, lymph node metastasis and extra-thyroidal extension (ETE) have been identified as predictors of recurrence and patient outcome (16–20). However, there has been considerable controversy regarding microscopic ETE (m-ETE) (as determined histologically using microscopic evaluation) and its prognostic significance, as well as its association with recurrence (21–23). The American Thyroid Association (ATA) guidelines for predicting recurrence considers patients with m-ETE to be at an intermediate risk for recurrence (6), suggesting that these patients should be treated with more aggressive treatment and radioiodine (RAI) ablation. In agreement with this, the European Thyroid Association is also in favor of RAI ablation for PTC patients presenting with m-ETE (24).

Although ATA guidelines are widely used, the American Joint Committee on Cancer (AJCC) TNM staging remains the most commonly used system for PTC staging. The most recent edition (8^th^) of AJCC staging system has removed m-ETE from the definition of pT3 disease, and minimized the clinical impact of m-ETE, down staging it from T3 to T1/2 classification compared to the 7^th^ edition (25). These changes reflect doubts on the ability to accurately identify m-ETE by histopathologist and its clinical prognostic impact in PTC. Whether the presence of m-ETE directly impacts clinical outcome and patient management is a matter of strong debate. While some studies have reported that m-ETE does not affect the disease free survival or risk of recurrence (22, 26–28), others report a negative impact on the clinical outcome of PTCs with m-ETE (21, 29, 30).

However, none of the above studies were conducted on PTCs from Middle Eastern ethnicity and the clinico-pathological associations as well as prognostic impact of m-ETE still remains unknown in this ethnicity. Therefore, we carried out this study to investigate the incidence and clinical impact of m-ETE as predictor of patient’s prognosis in Middle Eastern PTCs treated at our institute whilst also comparing the prognostic performance of AJCC 7^th^ (pT-7) and AJCC 8^th^ (pT-8) edition T staging systems.

## Materials and Methods

### Patient Selection

One thousand four-hundred and thirty consecutive unselected adult PTC patients (> 18 years) diagnosed between 1988 and 2018 at King Faisal Specialist Hospital and Research Centre (Riyadh, Saudi Arabia) were included in the study. Cases were identified based on clinical history followed by fine needle aspiration cytology for confirmation. The Institutional Review Board of the hospital approved this study and the Research Advisory Council (RAC) provided waiver of consent under project RAC # 2110 031 and 2211 168.

### Clinico-Pathological Data

Baseline clinico-pathological data were collected from case records and have been summarized in **Table 1**. Extra-thyroidal extension was further classified, based on previous publications (31–33), as follows: microscopic ETE was defined as tumor extending beyond the thyroid capsule into the surrounding peri-thyroidal soft tissues of fat and/or skeletal muscle, without visual evidence of this invasion and macroscopic ETE defined as visual evidence of tumor invasion into strap muscles, subcutaneous soft tissue, larynx, trachea, esophagus, recurrent laryngeal nerve or prevertebral fascia. Staging of PTC was performed using the AJCC seventh and eighth edition staging systems. Only structural recurrence (local, regional or distant) was considered for analysis. Recurrence was defined as any newly detected tumor or metastatic lymph node based on ultrasound and/or imaging studies in patients who had been previously free of disease following initial treatment. RAI refractory disease and ATA risk categories were defined based on 2015 ATA guidelines (6).

**Table 1:** Patient characteristics of the PTC cohort.

	Total
	No. (%)
**Total**	1430
**Age at surgery (years)**	
Median	39.2
Range	18.0 – 87.6
**Gender**	
Male	345 (24.1)
Female	1085 (75.9)
**Histologic subtype**	
Classical variant	943 (65.9)
Follicular variant	260 (18.2)
Tall cell variant	132 (9.2)
Other variants	95 (6.7)
**Tumor size (cm)**	
≤ 1	207 (14.5)
1 – 2	419 (29.3)
2 – 4	516 (36.1)
>4	288 (20.1)
**Tumor laterality**	
Unilateral	984 (68.8)
Bilateral	446 (31.2)
**Tumor focality**	
Unifocal	733 (51.3)
Multifocal	697 (48.7)
**Lymphovascular invasion**	
Present	296 (20.7)
Absent	1134 (79.3)
**Extrathyroidal extension (ETE)**	
No ETE	878 (61.4)
Micro-ETE	446 (31.2)
Macro-ETE	106 (7.4)
**AJCC 8 pathologic T stage**	
pT1a	192 (13.4)
pT1b	406 (28.4)
pT2	474 (33.1)
pT3a	252 (17.6)
pT3b	15 (1.1)
pT4a	91 (6.4)
pT4b	0
**Pathologic N stage**	
N0	616 (43.1)
N1	692 (48.4)
Nx	122 (8.5)
**Distant metastasis**	
M0	1361 (95.2)
M1	69 (4.8)
**RAI Refractory**	
Yes	235 (19.8)
No	953 (80.2)
**Recurrence**	
Yes	260 (18.2)
No	1170 (81.8)
***BRAF* mutation**	
Present	776 (57.2)
Absent	580 (42.8)

### *BRAF* Mutation Analysis

*BRAF* mutation data for the entire PTC cohort was available from our previous study (34).

### Follow-Up and Study Endpoint

Patients were regularly followed by both physical examinations and imaging studies to identify tumor recurrence. The median follow-up was 9.3 years (range 1.0 – 30.1 years). The primary study endpoint for our analysis was recurrence-free survival (RFS). RFS was defined as the time (in months) from date of initial surgery to the occurrence of any tumor recurrence (local, regional or distant). In case of no recurrence, date of last follow-up was the study endpoint.

### Statistical Analysis

The associations between clinico-pathological variables and extrathyroidal extension was performed using contingency table analysis and Chi square tests. Mantel-Cox log-rank test was used to evaluate recurrence-free survival. Survival curves were generated using the Kaplan-Meier method. Cox proportional hazards model was used for multivariate analysis. Two-sided tests were used for statistical analyses with a limit of significance defined as p value < 0.05. Data analyses were performed using the JMP11.0 (SAS Institute, Inc., Cary, NC) software package.

The relative prognostic performance of each T staging system was evaluated using the Proportion of Variation Explained (PVE) and Harrell’s Concordance Index (C-index). The PVE in Cox-proportional hazard model was calculated to compare the relative validity of models with AJCC 7^th^ and 8^th^ T stages. The PVE ranges from 0% to 100%, with a higher number indicating better predictability (35). Additionally, we evaluated the predictive capacity of the two models using the Harrell’s C-index. It is commonly used to evaluate risk models in survival analysis (36, 37). A model with perfect predictive capacity (sensitivity and specificity of 100%) would have a Harrell’s C-index of 1·00; a category that exhibited a higher Harrell’s c-index was considered to exhibit a more accurate predictive capacity. C-index and PVE were calculated using R version 4.0.1.

## Results

### Patient and Tumor Characteristics

Median age of the study population was 39.2 years (range: 18 – 88 years), with a male to female ratio of 1:3. The majority of tumors were classical variant of PTC (65.9%; 943/1430). 31.2% (446/1430) of tumors were bilateral and 48.7% (697/1430) were multifocal. m-ETE was noted in 31.2% (446/1430), whereas 7.4% (106/1430) of tumors showed macroscopic ETE. Tumor recurrence was seen in 18.2% (260/1430) (**Table 1**).

### Clinico-Pathological Associations of Microscopic Extrathyroidal Extension

**Figure 1:**
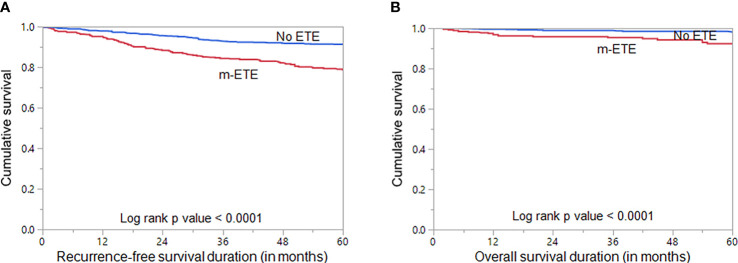
Survival Analysis of microscopic extrathyroidal extension (m-ETE). Kaplan Meier survival plot showing statistically significant poor **(A)** recurrence-free survival (p < 0.0001) and **(B)** overall survival (p < 0.0001) in PTC patients with m-ETE compared to those with no ETE.

We examined the clinico-pathological associations of m-ETE in our cohort. For this purpose, we excluded patients with macroscopic ETE (n = 106). Of the remaining 1324 PTCs, 33.7% (446/1324) showed m-ETE, whereas 66.3% (878/1324) had no ETE. m-ETE was significantly associated with adverse clinico-pathological characteristics such as age ≥ 55 years (p < 0.0001), male sex (p = 0.0245), tall cell variant (p < 0.0001), bilateral tumors (p < 0.0001), multifocality (p < 0.0001), lymphovascular invasion (p < 0.0001), regional lymph node metastasis (p < 0.0001), distant metastasis (p = 0.0166), poor RAI response (p < 0.0001) and tumor recurrence (p < 0.0001). We also found a significant association between m-ETE and *BRAF* mutation (p < 0.0001) (**Table 2**). Furthermore, patients exhibiting m-ETE showed a significantly reduced RFS (p < 0.0001) (**Figure 1A**). Since the AJCC 8^th^ classification preferred tumor size over m-ETE for classifying patients as T3a, we sought to determine whether tumor size or m-ETE was an independent predictor of RFS. On multivariate analysis, m-ETE was an independent predictor of RFS (HR = 1.75; 95% CI = 1.30 – 2.35; p < 0.0001), irrespective of tumor size (**Table 3**). We also analyzed the overall survival (OS) and found that m-ETE was associated with poor OS only on univariate analysis (p < 0.0001) (**Figure 1B**) but not on multivariate analysis (HR = 1.73; 95% CI = 0.82 – 3.77; p = 0.1503).

**Table 2:** Clinico-pathological associations of microscopic extrathyroidal extension (m-ETE) in PTC.

	Total		m-ETE present		No ETE		p value
	No.	%	No.	%	No.	%	
**Total**	1324		446	33.7	878	66.3	
**Age at surgery (years)**							
< 55	1083	81.8	325	72.9	758	86.3	< 0.0001
≥ 55	241	18.2	121	27.1	120	13.7	
**Gender**							
Male	313	23.6	122	27.4	191	21.8	0.0245
Female	1011	76.4	324	72.6	687	78.2	
**Histologic subtype**							
Classical variant	864	65.3	314	70.4	550	62.6	< 0.0001
Follicular variant	252	19.0	34	7.6	218	24.8	
Tall cell variant	116	8.8	73	16.4	43	4.9	
Other variants	92	6.9	25	5.6	67	7.6	
**Tumor laterality**							
Unilateral	926	69.9	280	62.8	646	73.6	< 0.0001
Bilateral	398	30.1	166	37.2	232	26.4	
**Tumor focality**							
Unifocal	688	52.0	193	43.3	495	56.4	< 0.0001
Multifocal	636	48.0	253	56.7	383	43.6	
**Lymphovascular invasion**							
Present	270	20.4	136	30.5	134	15.3	< 0.0001
Absent	1054	79.6	310	69.5	744	84.7	
**Regional lymph node metastasis**							
N0	589	48.8	119	29.0	470	59.1	< 0.0001
N1	617	51.2	292	71.0	325	40.9	
**Distant metastasis**							
Absent	1281	96.7	424	95.1	857	97.6	0.0166
Present	43	3.3	22	4.9	21	2.4	
**RAI Refractory**							
Yes	194	17.7	105	26.7	89	12.7	< 0.0001
No	900	82.3	288	73.3	612	87.3	
**ATA risk category**							
Intermediate	500	45.9	215	48.2	285	44.3	0.1980
High	590	54.1	231	51.8	359	55.7	
**Recurrence**							
Yes	213	16.1	119	26.7	94	10.7	< 0.0001
No	1111	83.9	327	73.3	784	89.3	
***BRAF* mutation**							
Present	701	55.9	311	72.7	390	47.3	< 0.0001
Absent	552	44.1	117	27.3	435	52.7	

**Table 3:** Multivariate analysis of microscopic extrathyroidal extension using Cox Proportional Hazard Model for Recurrence-free survival.

Clinico-pathological variables	Recurrence-free survival		
	Hazard ratio	95% Confidence interval	p-value
**Age**			
≥ 55 years (*vs*. < 55 years)	2.34	1.70 – 3.23	< 0.0001
**Sex**			
Male (*vs*. Female)	0.66	0.49 – 0.89	0.0060
**Histology**			
Tall cell variant (*vs*. other variants)	0.60	0.34 – 1.04	0.0070
**Tumor laterality**			
Bilateral (*vs*. Unilateral)	1.00	0.74 – 1.36	0.9800
**Tumor focality**			
Multifocal (*vs*. Unifocal)	1.00	0.76 – 1.33	0.9860
**Lymphovascular invasion**			
Present (*vs*. Absent)	0.91	0.63 – 1.32	0.6280
**Tumor size**			
≤1cm	Reference	0.43 – 1.17	0.1760
1-2cm	0.71	0.64 – 1.63	0.9230
2-4cm	1.02	0.99 – 2.58	0.0550
>4cm	1.60		
**Lymph node metastasis**			
Present (*vs*. absent)	2.23	1.63 – 3.05	< 0.0001
**Distant metastasis**			
Present (*vs*. absent)	5.44	3.49 – 8.48	< 0.0001
**Extrathyroidal extension (ETE)**			
Micro-ETE (*vs*. No ETE)	1.75	1.30 – 2.35	< 0.0001

### Prognostic Performance of AJCC 7 and 8 Classifications

Of 611 patients with T3 disease based on AJCC 7^th^ edition classification, 359 (58.8%) were down-staged in the AJCC 8^th^ edition classification. Among the 359 T3 patients who were down-staged in AJCC8 classification, 166 patients were downstaged to T1 and 193 patients were downstaged to T2. Twenty-two percent (79/359) of the downstaged patients developed tumor recurrence. Recurrence was noted in 18.7% (31/166) of patients who were downstaged from T3 to T1 and in 24.2% (48/193) of patients who were downstaged from T3 to T2 (**Table 4**). Since the proportion of recurrence in downstaged patients was higher than the overall cohort (22.0% *vs*. 18.2%), we next analyzed the prognostic performance of AJCC pT-7 and AJCC pT-8 classifications. Overall, the prognostic performance of pT-8 was inferior to pT-7 on the basis of lower PVE (3.04% *vs* 3.73%) and lower C-index (0.40 *vs* 0.48) (**Table 5**).

**Table 4:** Pathological tumor stage migration of AJCC 7^th^ edition T3 tumors and incidence of recurrence.

AJCC 8^th^ edition	AJCC 7^th^ edition	Recurrence
	T3, n (%)	n (%)
T1	166	31 (18.7)
T2	193	48 (24.2)
T3a	252	68 (27.0)
Total	611	147 (24.1)

**Table 5:** Comparison of Recurrence-free survival according to AJCC 7^th^ and 8^th^ edition T staging.

Variable	HR (95% CI)	P value	PVE (%)	C-index
Overall study cohort (Adult PTC)				
AJCC 7^th^ edition T stage			3.73	0.48
T1b *vs* T1a	0.97 (0.50 – 1.89)	0.930		
T2 *vs* T1a	1.04 (0.54 – 1.98)	0.910		
T3 *vs* T1a	2.96 (1.71 – 5.12)	0.001		
T4a *vs* T1a	6.60 (3.64 – 11.96)	0.001		
AJCC 8^th^ edition T stage			3.04	0.40
T1b *vs* T1a	1.31 (0.91 – 1.88)	0.141		
T2 *vs* T1a	1.36 (0.96 – 1.94)	0.088		
T3a *vs* T1a	1.01 (0.63 – 1.63)	0.959		
T3b *vs* T1a	2.24 (1.48 – 3.40)	0.001		
T4a *vs* T1a	2.86 (1.90 – 4.29)	0.001		

HR, Hazard ratio; CI, Confidence interval; PVE, Proportion of Variation Explained; PTC, Papillary Thyroid Carcinoma; AJCC, American Joint Committee on Cancer.

## Discussion

In the era of personalized medicine and risk-tailored management, risk stratification is necessary to provide appropriate therapy and predict response to the initial treatment. Recent research has indicated that m-ETE only exerts minor effect on patient prognosis and therapy decisions (28, 38, 39). The updated 8^th^ edition of the AJCC staging system removed the sub classification of m-ETE, resulting in down staging of T3 tumors. Therefore, we conducted this study to evaluate, for the first time, the clinico-pathological characteristics and clinical impact of m-ETE on patient outcome in a large cohort of Middle Eastern PTC.

In our study, the overall incidence of m-ETE was 31.2% in PTCs. Interestingly, m-ETE was associated with several adverse clinico-pathological factors such as older age group, tall cell variant, multifocality, lymphovascular invasion, regional lymph node metastasis, and radioiodine therapy refractiveness. Moreover, strong correlation between the presence of m-ETE and *BRAF* mutation was noted, with 72.7% of m-ETE PTC having concurrent *BRAF* mutations.

There is considerable controversy regarding the prognostic role of m-ETE. We found m-ETE to be associated with poor OS in univariate analysis only. Previous studies have found contrasting results, with some showing an unfavorable impact of m-ETE on OS (33, 40), whereas others found no association (28). Interestingly, in the present research, m-ETE was associated with higher risk of recurrence compared to PTCs without m-ETE and multivariate analysis revealed that m-ETE was an independent prognostic marker for poor RFS. This raises the likelihood that patients presenting with m-ETE might have more aggressive disease and are therefore less likely to respond well to initial treatment. With a median follow-up of 9.3 years, the present study revealed more recurrent disease among patients with m-ETE. Indeed, we showed that the presence of m-ETE conferred a 2.5-fold increased risk of recurrence for all ages (p < 0.0001; **Table 2**). Several previous attempts to identify the prognostic impact of m-ETE on patient outcome have shown conflicting results. Park et al. (41) analyzed a cohort of 734 PTC patients and found that m-ETE was an important prognostic factor associated with RFS. Similar studies by Radowsky et al. (21) and Tran et al. (42), on large cohorts of PTC, also showed that m-ETE was associated with recurrence and RFS. In contrast, other studies failed to find any prognostic impact for m-ETE. Nixon et al. (22) studied 984 patients (115 with m-ETE and 869 without m-ETE) and found no significant difference in 10-year disease-specific survival or RFS between the two groups. Hay et al. (38), Arora et al. (43) and Shin et al. (44), in large cohorts of PTC, also found no difference in RFS between patients with m-ETE and no ETE. Our results are the first to show that m-ETE is an independent predictor of poor RFS in this study population, irrespective of tumor size, and supports the inclusion of m-ETE in future AJCC T staging.

Although the AJCC staging system is primarily used as a predictor of mortality, recent studies have shown the utility of this staging system as a novel tool for predicting PTC recurrence (42, 45, 46). In addition, we were intrigued by the exclusion of m-ETE from the AJCC 8th edition staging system and hence sought to determine which of the two staging systems (AJCC 7 or AJCC 8 T stage) was a better predictor of recurrence. Therefore, we further compared the prognostic performance of the AJCC 7^th^ and 8^th^ edition staging system in the whole cohort and found the ability of pT-7 to predict RFS was superior to pT-8 based on different models’ performance regardless of age or the presence of distant metastasis. The superiority of the prognostic impact of pT-7 and pT-8 was highlighted in a previous study (42), where however, it was seen only in PTC patients ≥ 55 years old without distant metastasis. Another important highlight of this study is the significant association between m-ETE and poor response to RAI, which could reflect the potential influence of m-ETE on treatment decision. However, this needs to be confirmed by further studies, since our data only showed this correlation on univariate analysis. Collectively, our results support the inclusion of m-ETE in risk stratification, as in previous AJCC TNM editions and the ATA risk of recurrence guidelines.

Despite the obvious strengths of our study, including the use of more than 1400 PTCs from a unique ethnicity, the presence of comprehensive clinical and follow-up data and the finding of m-ETE to be a robust independent prognostic factor for RFS, irrespective of tumor size, our study should be viewed in light of a few limitations. Our study was a retrospective and single center study, which could carry selection bias, and hence more prospective multicenter studies in Middle Eastern population are needed. Additionally, we do acknowledge that inter-observer variability for the interpretation of m-ETE is a source of debate (47). However, the histopathologic sections have been reviewed by at least two pathologists to minimize the inter-observer and intra-observer variability. Despite our efforts, we cannot deny the effect of inter-observer and intra-observer variability in our study. Therefore, our conclusions should be interpreted with caution.

In conclusion, our study shows that m-ETE plays an important role in PTC patients from Middle Eastern ethnicity. We found that m-ETE alone is associated with aggressive PTC markers and is an independent marker for poor RFS. Thus omitting minimal ETE from the definition of T3 disease could compromise patient care and management, resulting in these patients being less likely to undergo RAI therapy. Therefore, our results support the inclusion of m-ETE in risk stratification models, such as the AJCC 7^th^ edition and the ATA risk of recurrence guidelines, for Middle Eastern PTC.

## Data Availability Statement

The original contributions presented in the study are included in the article/supplementary material. Further inquiries can be directed to the corresponding author.

## Ethics Statement

The Institutional Review Board of King Faisal Specialist Hospital and Research Centre approved this study and the Research Advisory Council (RAC) provided waiver of consent under project RAC # 2110 031 and 2211 168. Written informed consent for participation was not required for this study in accordance with the national legislation and the institutional requirements.

## Author Contributions

SP and AS analyzed the clinical data, designed and wrote the manuscript. ZQ and KS performed statistical analysis. FD performed clinical data abstraction. SA-S and FA-D contributed samples and analyzed clinical data. KA-K designed, implemented the study, wrote and critically reviewed the manuscript. All authors contributed to the article and approved the submitted version.

## Conflict of Interest

The authors declare that the research was conducted in the absence of any commercial or financial relationships that could be construed as a potential conflict of interest.

## Publisher’s Note

All claims expressed in this article are solely those of the authors and do not necessarily represent those of their affiliated organizations, or those of the publisher, the editors and the reviewers. Any product that may be evaluated in this article, or claim that may be made by its manufacturer, is not guaranteed or endorsed by the publisher.
